# Elevation of n-3/n-6 PUFAs ratio suppresses mTORC1 and prevents colorectal carcinogenesis associated with *APC* mutation

**DOI:** 10.18632/oncotarget.12759

**Published:** 2016-10-19

**Authors:** Miao Liu, Ling Zhou, Baiyu Zhang, Minhong He, Xiaoying Dong, Xiaojun Lin, Chunhong Jia, Xiaochun Bai, Yifan Dai, Yongchun Su, Zhipeng Zou, Hang Zheng

**Affiliations:** ^1^ Department of Oncology, Nanfang Hospital, Southern Medical University, Guangzhou 510515, China; ^2^ Department of Rheumatology, the Sixth Affiliated Hospital of Sun Yat-Sen University, 510655, China; ^3^ Department of Cell Biology, School of Basic Medical Science, Southern Medical University, Guangzhou 510515, China; ^4^ State Key Laboratory of Reproductive Medicine and Jiangsu Key Laboratory of Xenotransplantation, Nanjing Medical University, Nanjing 201129, China; ^5^ Department of Bioinformatics, School of Basic Medical Science, Southern Medical University, Guangzhou 510515, China

**Keywords:** PUFA, mTOR complex 1, colorectal cancer, APC

## Abstract

Although epidemiological and preclinical studies have shown the preventative effect of n-3 polyunsaturated fatty acids (PUFAs) on colorectal cancer (CRC), the underlying molecular mechanisms are not clear. In this study, we revealed that elevation of n−3/n-6 PUFAs ratio suppress the mechanistic target of rapamycin complex 1 (mTORC1) and prevent colorectal tumorigenesis. The transgenic expression of *fat-1*, a desaturase that catalyzes the conversion of n-6 to n-3 PUFAs and produces n-3 PUFAs endogenously, repressed colorectal tumor cell growth and remarkably reduced tumor burden, and alleviated anemia as well as hyperlipidemia in *APC^Min/+^* (adenomatous polyposis coli) mice, a classic CRC model that best simulates most clinical cases. In contrast to arachidonic acid (AA, C20:4 n−6), either Docosahexaenoic acid (DHA, C22:6 n−3), eicosapentaenoic acid (EPA, C20:5 n−3), or a combination of DHA and AA, efficiently inhibited the proliferation of CRC cell lines and promoted apoptosis in these cells. The ectopic expression of fat-1 had similar effects in colon epithelial cells with APC depletion. Mechanistically, elevation of n−3/n−6 ratio suppressed mTORC1 activity in tumors of *APC^Min/+^* mice, CRC cell lines with *APC* mutation, and in normal colon epithelial cells with *APC* depletion. In addition, elevation of n−3/n−6 ratio repressed mTORC1 activity and inhibited adipogenic differentiation in preadipocytes with *APC* knockdown, as well as alleviated hyperlipidemia in *APC^Min/+^* mice. Taken together, our findings have provided novel insights into the potential mechanism by which increase in n−3/n−6 PUFAs ratio represses CRC development, and also a new rationale for utilizing n-3 PUFAs in CRC prevention and treatment.

## INTRODUCTION

Colorectal cancer (CRC) ranks the second most prevalent cancer among women and the third among men worldwide [1]. Crucial risk factors have been identified by epidemiological studies, including but not limited to the imbalanced dietary intake of fatty acids, high concentration of serum triglycerides and cholesterol, and mutations in key tumor suppressor genes such as *adenomatous polyposis coli* (*APC*). Notably, since *APC* is an endogenous inhibitor of tumorigenic β-catenin, the *APC* mutation leads to β-catenin stabilization, constitutive activation of Wnt signaling, and the consequent familial adenomatous polyposis (FAP) and sporadic colorectal cancer [2–5]. As attractive candidate ‘natural’ CRC chemoprevention agents, n-3 polyunsaturated fatty acids (PUFAs) have demonstrated beneficial effects in the prevention of CRC in both humans and mice. A case control study of 1872 patients demonstrated a significant dose-dependent reduction in CRC risk for total n-3 PUFAs intake, as well as for EPA and DHA intake individually [6]. Moreover, a Phase III double-blind randomized clinical trial with EPA for 6 months on rectal polyps in patients with FAP provided the first definitive evidence of chemopreventive efficacy of EPA in humans with a marked decrease in adenoma numbers and a cumulative reduction in adenoma size [7]. Consistently, rodents fed n-3 PUFAs versus n−6 PUFAs or low-fat control diet controls showed a 20–50% reduction in tumor incidence induced by both *APC* mutation and carcinogens [8]. However, the underlying mechanisms associated with this effect remain unclear.

Mechanistic target of rapamycin (mTOR) is a highly conserved Ser/Thr kinase that integrates diverse components, including nutrients, growth factors, energy, and stresses, to control cell growth, proliferation, survival and metabolism [9–13]. mTOR elicits its pleiotropic functions via two functionally distinct signaling complexes known as mTOR complex 1 (mTORC1) and complex 2 (mTORC2). mTORC1 plays a key role in translation initiation by directly phosphorylating p70 S6 kinase 1 (S6K1) and 4E-BP1. Recent evidence indicates that mTORC1 signaling is a key event in promoting the development of colorectal cancer, and the activation of the mTORC1 signaling pathway is observed in up to 40% cases of colorectal tumor [13–18]. Considering that mTORC1 promotes adipocyte differentiation [19] as well as lipid uptake and synthesis in adipose tissue [20], and considering our previous study on the targeting of mTORC1 by n−3 PUFAs in breast cancer [21], we hypothesize that n−3 PUFAs suppress mTORC1 activity and thereby prevent CRC and associated hyperlipidemia.

Because the amount, source, and ratio of PUFAs vary widely and lead to inconsistent results, a transgenic mouse model that expresses *fat-1*, an n−3 fatty acid desaturase, was developed [22]. Since this enzyme can catalyze the conversion of n-6 PUFAs to n−3 PUFAs by introducing a double bond into fatty acyl chains, the *fat-1* transgenic mice will enable the investigation of the biological properties of n-3 PUFAs without having to incorporate n−3 PUFAs in the diet, and this strategy represents a more reliable and intuitive model [23]. Since mutations in the gene coding for *APC* account for most clinical cases of familial adenomatous polyposis (FAP) and sporadic colonic tumors [5], C57BL/6J-*APC*^Min/+^ mouse strains have been established to simulate CRC associated with *APC* mutation, with multiple intestinal adenomas and complications that resemble clinical CRC cases, including anemia and rectal prolapse [24, 25].

In this study, for the first time we crossed *fat-1* transgenic mice with *APC*^Min/+^ mice and generated a novel mouse strain, *fat-1-APC*^Min/+^ double-hybrid, to investigate the preventive effects of elevation of n–3/n–6 ratio on CRC associated with *APC* mutation and the underlying mechanisms. Elevated n−3/n−6 ratio suppressed the activity of mTORC1 *in vitro* and *in vivo*, prevented colorectal carcinogenesis and tumor growth, and alleviated associated anemia and rectal relapse. Taken together, our results provide a new rationale and exciting therapeutic value of utilizing n–3 PUFAs to target CRC and associated anemia, particularly in cases involving *APC* mutation.

## RESULTS

### *fat-1- APC^Min/+^* mice demonstrate marked suppression of colorectal tumorigenesis and accompanying anemia compared with *APC^Min/+^* mice

**Figure 1:**
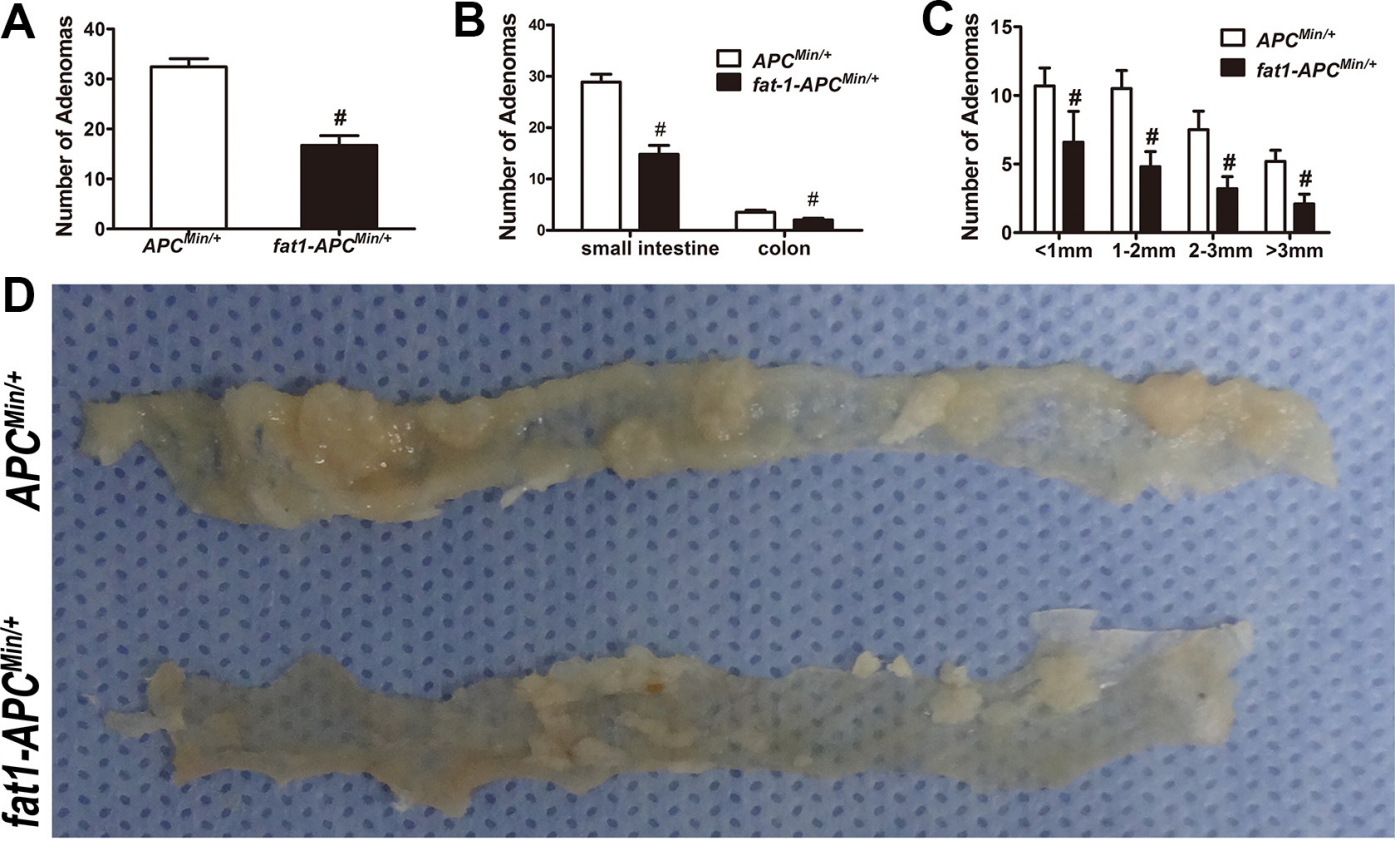
*Fat-1-APC^Min/+^* mice manifest a clear reduction in the number and size of adenomas compared with *APC^Min/+^* mice *APC^Min/+^*-*fat-1* mice (*n* = 10) and control *APC*^Min/+^ mice (*n* = 10) from the same litter were killed for colorectal tumorigenesis analysis. Number of adenomas in total (**A**) and in small intestine or colon, respectively (**B)** were shown. (**C**) Average tumor burden mass of all colorectal tumors counted in each *fat-1*-*APC^Min/+^* and *APC^Min/+^* mouse. Bars indicate mean ± SEM; ^#^*P* < 0.05. (**D**) Representative images of tumors from each group.

**Figure 2:**
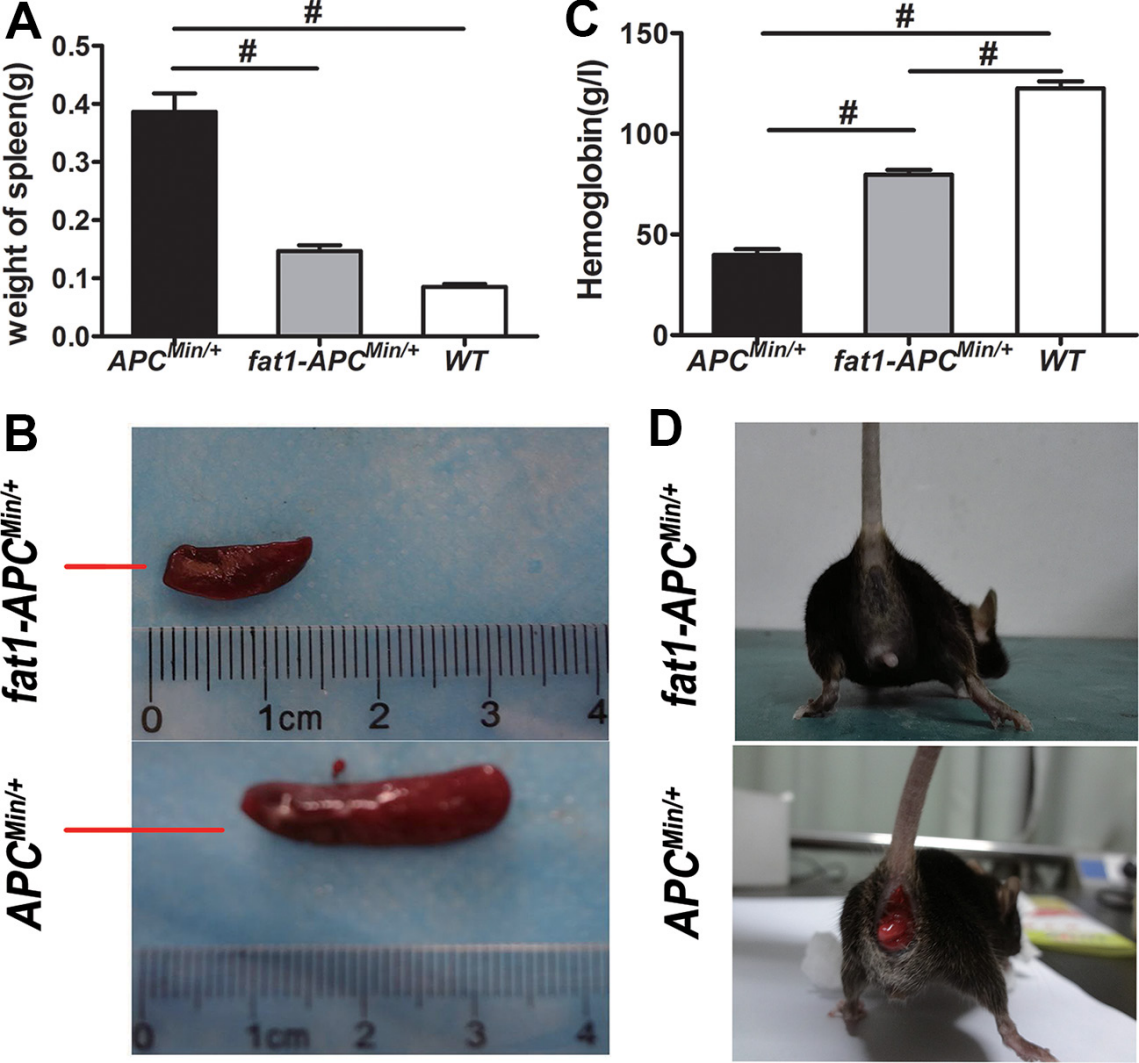
Anemia is ameliorated and rectal prolapse is prevented in *fat-1*-*APC*^Min/+^ mice compared with APC^Min/+^ mice (**A**) Spleen weight, (**B**) representative images of spleen, and (**C**) the level of hemoglobin in *fat-1*-*APC*^Min/+^ mice (*n* = 6), *APC*^Min/+^ mice (*n* = 6), and WT mice (*n* = 6). Bars indicate mean ± SEM; ^#^*P* < 0.05. (**D**) Presence or absence of rectal prolapse in *APC*^Min/+^ mice and *fat-1*-*APC*^Min/+^ mice, respectively.

The *fat-1* transgenic mice were crossed with the *APC*^Min/+^ mice to create a novel double-hybrid mice model with the view to provide a purely genetic approach to investigate the effects of elevation of n−3/n−6 PUFA ratio on colorectal tumorigenesis. The generation of *fat-1-APC*^Min/+^ mice was not only verified through genotyping (Supplementary Figure S1) but also confirmed by examining the relative content of n-6 and n-3 PUFAs in the tails (Supplementary Table S1) as well as in mucosal tissues (Supplementary Table S2). In both tissue samples of the *fat-1-APC*^Min/+^ mice there was a significant elevation of n-3/n-6 PUFAs ratio compared with that of *APC*^Min/+^ control mice. As expected, the number of tumors palpated in *fat-1-APC*^Min/+^ mice, whether in the small intestine or colon, was significantly lower (*P* < 0.05) than that in *APC*^Min/+^ control mice (Figure 1A–1C). In addition, the volume of the tumors in *fat-1-APC*^Min/+^ mice was much smaller than that in the *APC*^Min/+^ mice (Figure 1C and 1D), and anemia, one of the most common complications in clinical cases of CRC in *APC*^Min/+^ mice [26–28], was significantly mitigated in *fat-1-APC*^Min/+^ mice, on the basis of the spleen weight and hemoglobin level (Figure 2A–2C). Moreover, rectal prolapse, another complication of CRC, was completely prevented in *fat-1-APC*^Min/+^ mice (Figure 2D)

### Endogenous production or exogenous delivery of n-3 PUFAs suppresses the proliferation and promotes apoptosis in CRC cell lines and normal colon epithelia with *APC* depletion

**Figure 3:**
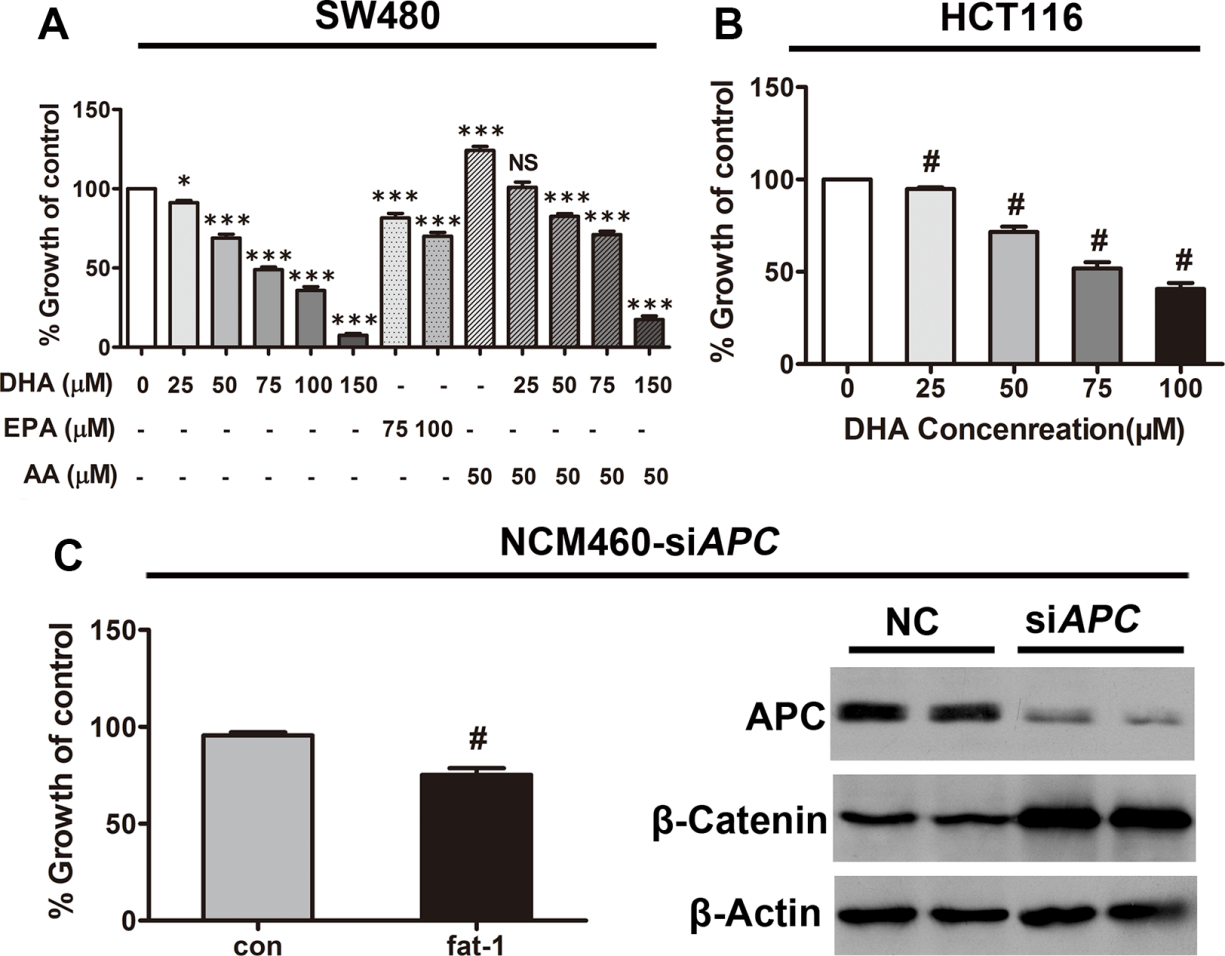
DHA and endogenous n-3 PUFAs repress the proliferation of colorectal tumor cells (**A**) SW480 and (**B**) HCT116 cells plated in 96-well plates were treated with serial concentrations of DHA (20–100 μM) for 72 h, with the replacement of DHA every 24 h. Cell viability was assessed using Cell Counting Kit-8. ^#^*p* < 0.01 compared with the group not treated with DHA. (**C**) NCM460 cells previously transfected with *APC* siRNA were transfected with the pST180-*fat-1* vector or with the control vector. After 48 h, cell viability was assessed as described above; ^#^*p* < 0.01 compared with the respective control. Bars indicate mean ± SEM.

To investigate the potential protective role of increasing n-3/n-6 ratio against CRC associated with *APC* mutation *in vitro*, we examined the effect of Docosahexaenoic acid (DHA, C22:6 n−3), eicosapentaenoic acid (EPA, C20:5 n-3), arachidonic acid (AA, C20:4 n−6), or a combination of both DHA or AA, on the proliferation of SW480, a CRC cell line with truncated *APC* and consequently with constitutively active β-catenin-TCF-regulated transcription (CRT, the best established hallmark of activated Wnt signaling), and on the proliferation of HCT116 cells, a CRC cell line with a β-catenin mutation that also leads to constitutively active CRT [29]. As expected, DHA or EPA dose-dependently inhibited cell proliferation in these cells (Figure 3A and 3B), which is consistent with our previous results in breast cancer cells [21]. Additionally, CRC cell proliferation promoted by AA was successfully inhibited by simultaneous treatment with DHA, in a dose-dependent manner (Figure 3A). Furthermore, we transfected NCM460 cells with the *APC* siRNA (designated NCM460-si*APC*s), because the loss of function of *APC* may be a crucial event in the early transformation of colonic epithelium [30], to interrogate the effect of increased n−3/n−6 ratio on the early transformed colonic epithelium. In consistence, the ectopic expression of *fat-1* in NCM460-si*APC*s also resulted in a significant elevation of n−3/n−6 ratio in these cells (Supplementary Table S3) suppressed their proliferation (Figure 3C left).

As expected, APC was sufficiently depleted, which leads to β-catenin stabilization (Figure 3C right).

**Figure 4:**
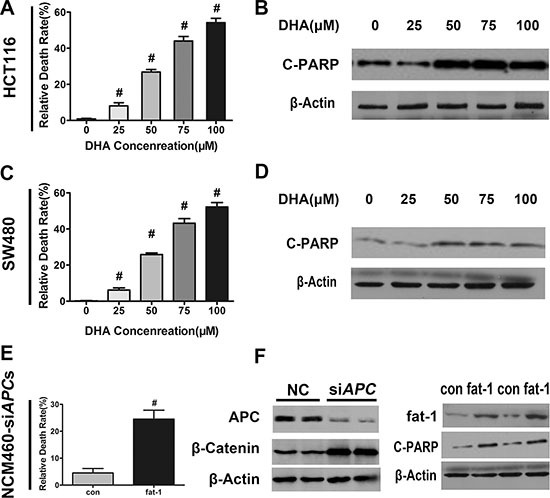
DHA promotes colorectal tumor cell apoptosis *in vitro* DHA promotes colorectal tumor cell apoptosis *in vitro*. (**A**) HCT116, (**C**) SW480, or (**E**) NCM460 cells transfected with *APC* siRNA were serum-starved and cultured with or without the indicated concentrations of DHA (dissolved in basic medium) for 12 h, and subsequently trypsinized to make single cell suspensions for trypan blue staining. Cell death was quantified using an automatic cell counter (BioRad) according to the manufacturer's instructions. Data from three repeat counts were averaged. Bars indicate mean ± SEM; ^#^*p* < 0.01. (**B**) HCT116, (**D**) SW480, or (**F**) NCM460 cells transfected with *APC* siRNA, serum-starved, and cultured for 12 h in the presence of the indicated concentrations of DHA. Proteins were extracted for the immunoblot analysis of cleaved-PARP (C-PARP).

Furthermore, studies have shown that n−3 PUFAs promote apoptosis in a variety of cancer cells [31]. We next examined whether n-3 PUFAs potentiate cellular apoptosis in CRC *in vitro*. DHA dose-dependently increased the number of dead cells in serum-starved HCT116, SW480, and NCM460-si*APC* cell lines (Figure 4A, 4C, and 4E). The cleavage of poly (ADP-ribose) polymerase (PARP) by active caspase-3—widely accepted as a hallmark of late-stage apoptosis but not necrosis—was consistently enhanced by DHA in a dose-dependent manner (Figure 4B, 4D, and 4F). Moreover, while AA alone had little effect, both DHA, EPA and a combination of DHA and AA markedly increased apoptosis of SW480 cells under serum starvation, as shown by Annexin V-FITC assay (Supplementary Figure S2A and S2B).

### Endogenous production or exogenous delivery of n-3 PUFAs inhibit mTORC1 activity in *APC^Min/+^* mice, CRC cell lines, and NCM460-si*APC* cells

**Figure 5:**
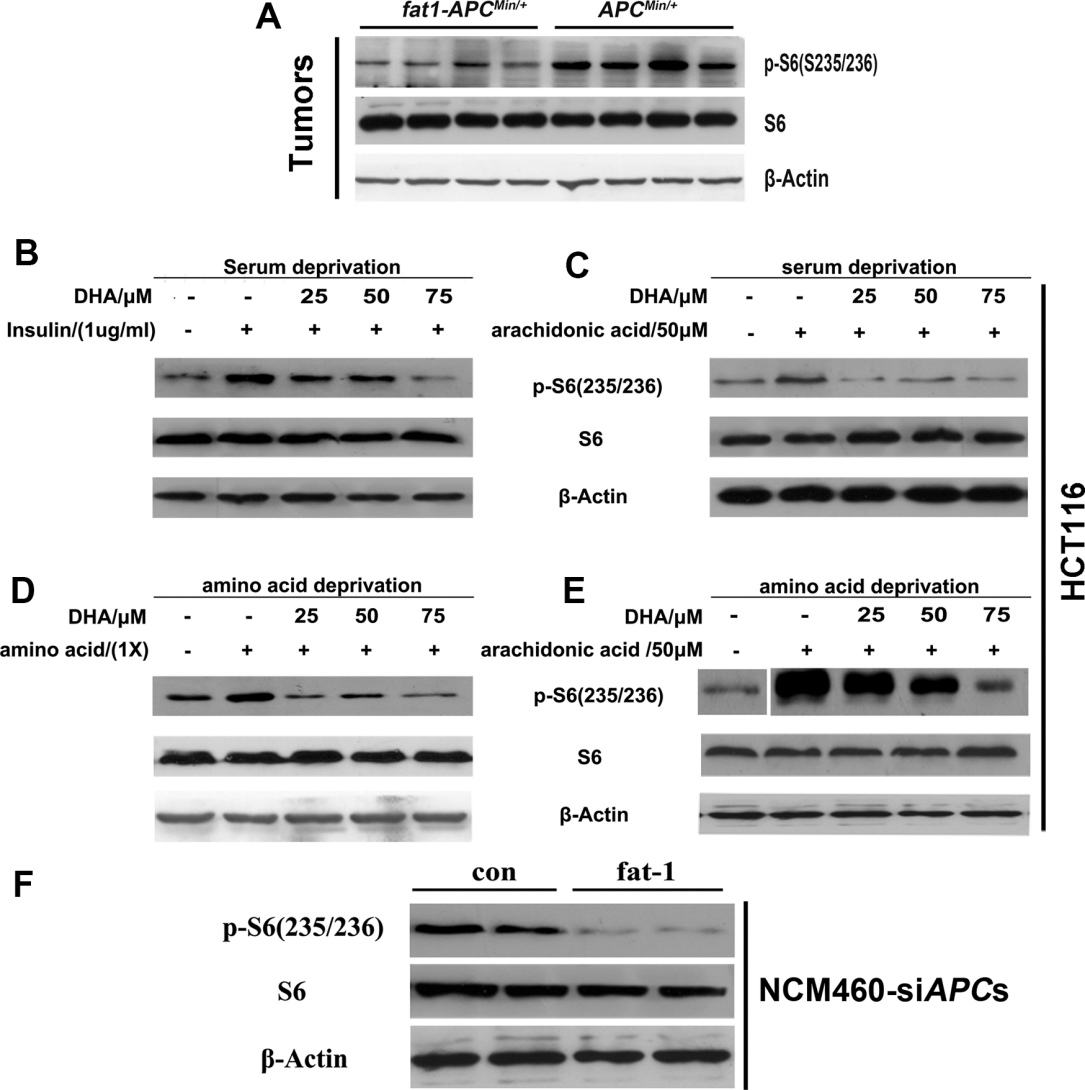
n-3 PUFAs inhibit mTORC1 signaling *in vivo* and *in vitro* (**A**) Tumor lysates were subjected to western blotting for measurement of the levels of phosphorylation of S6 (S235/236). HCT116 cells were serum-starved for 18 h, cultured in the presence of the indicated concentrations of DHA for 30 min, and stimulated with insulin (1 mg/ml) (**B**) or arachidonic acid (50 μM) (**C**) for 30 min. HCT116 cells were deprived of amino acids and incubated for 30 min in the presence of DHA, followed by incubation with amino acids (1×) (**D**) or arachidonic acid (50 μM) (**E**) for 30 min. The protein extract from each set was subjected to western blotting for the analysis of mTORC1 signaling. (**F**) NCM460 cells previously transfected with *APC* siRNA were transfected with a pST180 empty vector (con) or with the pST-180-*fat-1* vector (fat-1) and serum-starved for 18 h. The protein extract from each set was subjected to western blotting for the analysis of mTORC1 signaling.

Recent studies have revealed the critical role of mTORC1 activity in the proliferation and survival of CRC cells. Interestingly, our previous results demonstrated that arachidonic acid (AA), an n−6 PUFA, induced mTORC1 activity and promoted breast carcinogenesis and angiogenesis [32]. The opposite effects of n-3 and n-6 PUFAs in CRC prompted us to examine whether n-3 PUFAs negatively regulate mTORC1 signaling and consequently inhibit the proliferation and survival of CRC cells. Consistent with our hypothesis, the phosphorylation at S235/236 of S6 (the best characterized indicator of mTORC1 activation) was reduced in the tumors of *fat-1-APC*^Min/+^ mice compared with those of *APC*^Min/+^ control mice (Figure 5A). DHA rapidly suppressed the levels of insulin and amino acid-stimulated phosphorylation of S6 in a dose-dependent manner (S235/236) in HCT116 cells (Figure 5B–5E). Consistently, DHA or EPA significantly reduced phosphorylation of p70S6K1 (T389, another key hallmark of mTORC1 activation) induced by insulin and (or) AA treatment in SW480 cells under serum starvation (Supplementary Figure S3A), and to a lesser extent, under normal growth conditions (Supplementary Figure S3B). We next examined the effect of endogenously produced n−3 PUFAs on mTORC1 signaling. In NCM460-si*APC*s, *fat-1* overexpression significantly reduced the phosphorylation of S6 (S235/235) compared with the cells transfected with the control vector (Figure 5F).

### Inhibition of mTORC1 phenocopies the effects of n-3 PUFAs on proliferation and apoptosis of CRC cells

**Figure 6:**
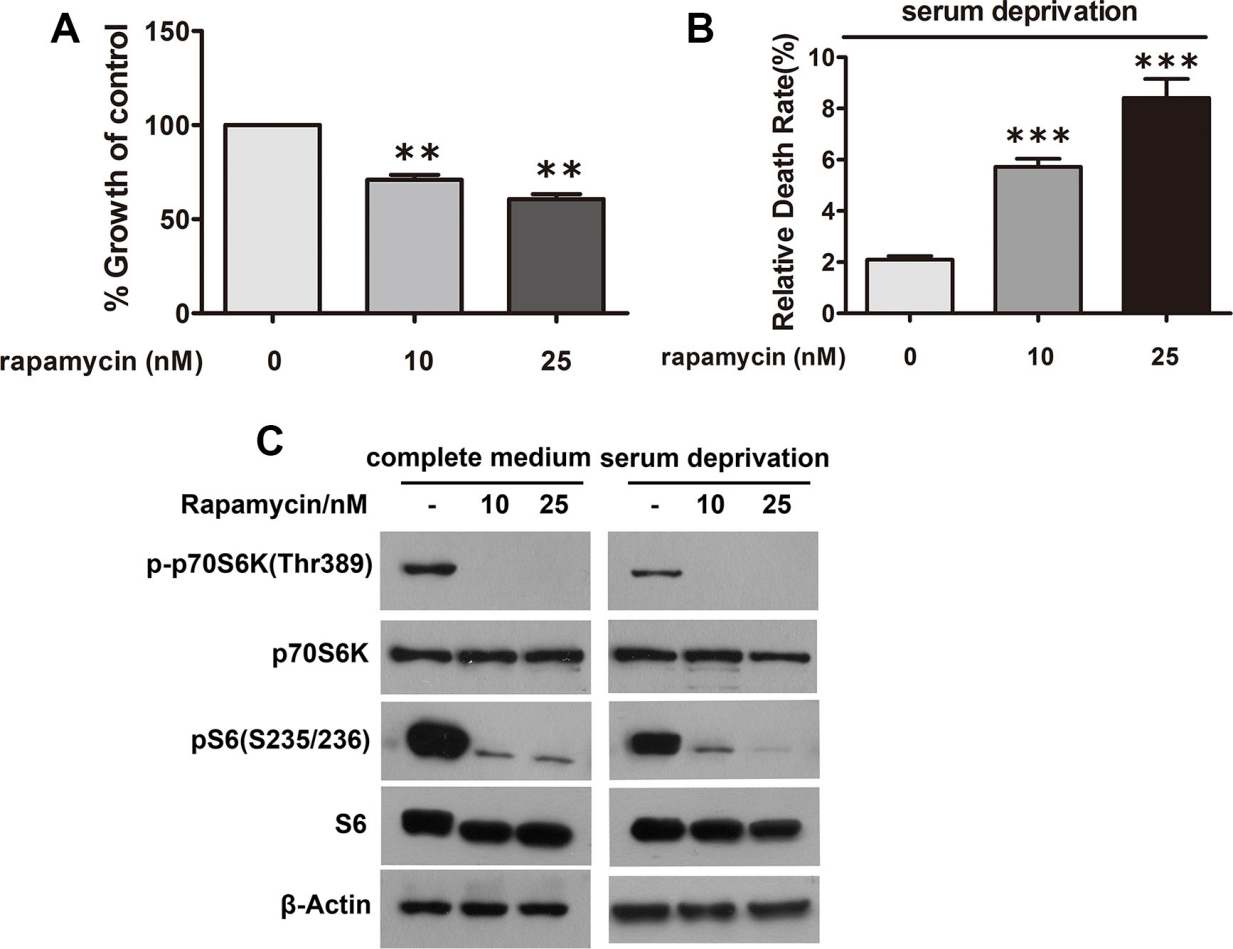
Inhibition of mTORC1 signaling in CRC cells has growth inhibitory and apoptotic effects similar to n-3 PUFAs (**A**) SW480 cells under normal growth conditions were treated with rapamycin as indicated and assessed for cell proliferation using Cell Counting Kit-8. **p < 0.01 VS control. (**B**) SW480 cells under serum deprivation were treated as in (A) and analyzed for cell death via trypan blue staining. ***p < 0.0001 VS control. (**C**) Verification of mTORC1 inhibition in (A) and (B) by visualization of S6 and p70S6K1 phosphorylation.

To interrogate whether the effects of n-3 on proliferation and apoptosis of CRC cells are through mTORC1, SW480 cells treated with rapamycin, a specific mTORC1 inhibitor, were analyzed for proliferation or viability, respectively. As expected, rapamycin treatment suppressed proliferation of SW480 cells (Figure 6A) and enhanced their death (Figure 6B), phenocopying that of n-3 PUFAs and suggesting n−3 PUFAs' effects on CRC cells are via mTORC1, at least partially.

### Ectopic expression of *fat-1* reduces mTORC1 activity and suppresses adipogenic differentiation in preadipocytes with silenced *APC*, and *fat-1* transgenic *APC^Min/+^* mice have lower serum lipid levels

**Figure 7:**
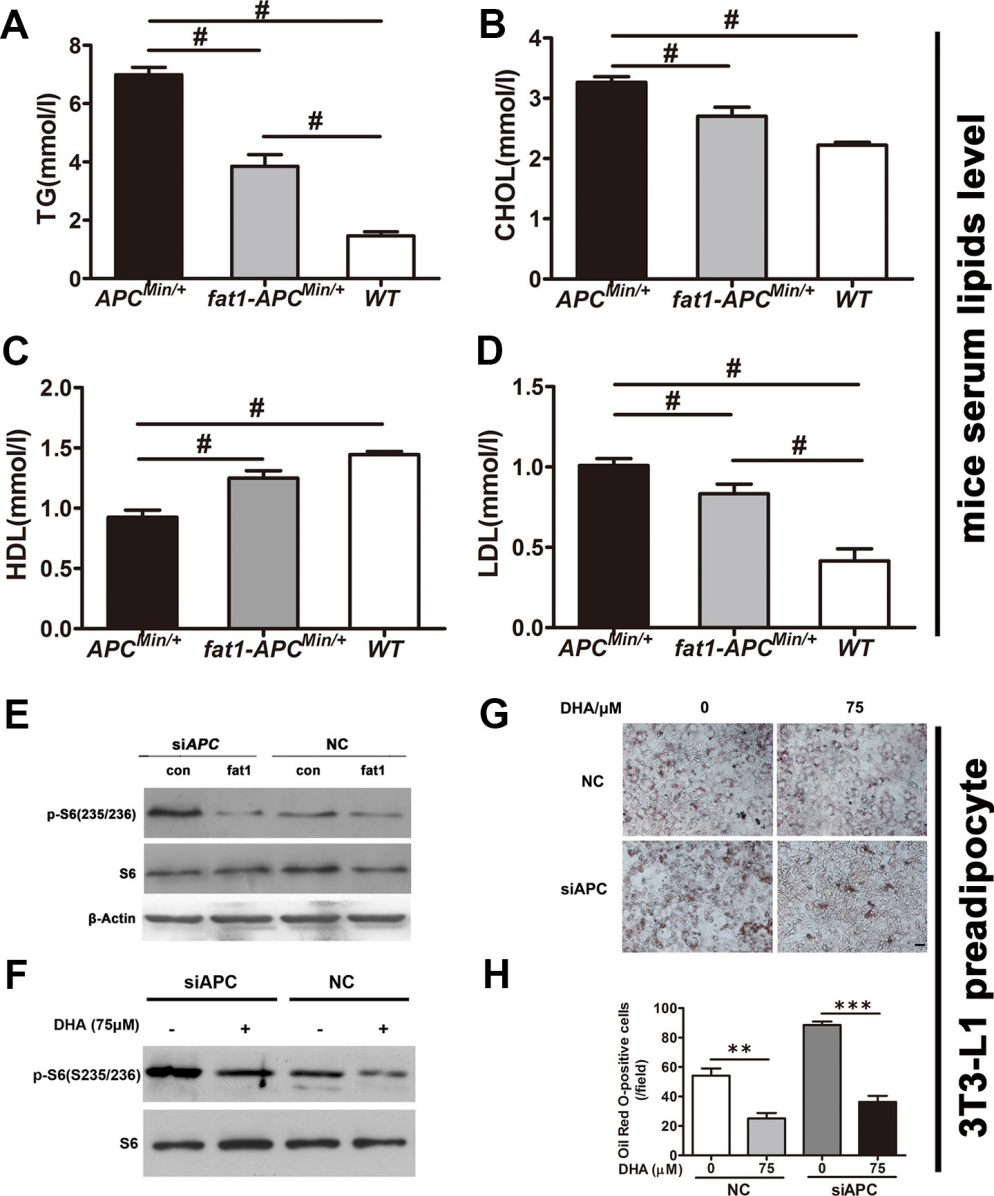
n-3 PUFAs inhibit serum lipids in *APC*^*Min/+*^ mice and suppress differentiation preadipocytes with silenced *APC* Blood samples were collected by heart puncture, kept at room temperature for 2 h for coagulation, centrifuged at 3,000× g for 15 min, and used for the detection of blood lipids. The levels of TG (**A**), CHOL (**B**), HDL (**C**), and LDL (**D**) in *fat-1*- *APC^Min/+^* mice (*n* = 6) were compared with those in *APC^Min/+^* mice (*n* = 6). Bars indicate mean ± SEM; ^#^*P* < 0.05. (**E**) 3T3-L1 cells previously transfected with *APC* siRNA were further transfected with a pST180 empty vector (con) or with the pST-180-*fat-1* vector (fat-1). The cells were harvested and the protein extract from each set was subjected to western blotting for the analysis of mTORC1 activity. (F) 3T3-L1 cells previously transfected with *APC* siRNA were treated with or without DHA and analyzed for mTORC1 activity. (**G**) 3T3-L1 cells transfected as in (**F**) were induced to differentiate into adipocytes with the differentiation medium supplemented with or without DHA. At 8 days post-transfection, cells were fixed, stained with Oil Red O, and visualized by bright-field microscopy. Scale bar, 50 μM. (**H**) Statistical analysis of Oil Red O positive cells as indicated. **p < 0.01, ***p < 0.0001.

Accompanying the markedly reduced mTORC1 activity, *fat-1*-*APC*^Min/+^ mice demonstrated significantly decreased serum lipid levels compared with *APC*^Min/+^ mice (Figure 7A–7D). Since mTORC1 activity is critical for the terminal differentiation of 3T3-L1 preadipocytes [19], we analyzed the effects of ectopic *fat-1* expression or DHA treatment on mTORC1 activity and adipogenic differentiation in 3T3-L1 with previous depletion of the *APC* gene. As expected, *APC* depletion triggered a marked induction of both mTORC1 activity (based on the observation of the phosphorylation of S6), which was considerably reversed by either overexpression of *fat-1* (Figure 7E) or DHA treatment (Figure 7F). Consistently, adipogenic differentiation induced by *APC* depletion in 3T3-L1 preadipocytes was markedly reversed by DHA treatment (Figure 7G and 7H). Collectively, these results suggest that the cellular productions of n-3 PUFAs and lowered n−6/n−3 ratio effectively alleviates hyperlipidemia, which is closely correlated with CRC associated with *APC* mutation, possibly via suppression of mTORC1 activity in adipogenesis.

## DISCUSSION

Previous studies have found that n−3 PUFAs are beneficial for the prevention and treatment of CRC [8]. However, the mechanisms underlying n-3 PUFAs' effects on CRC, especially CRC due to *APC* mutations, which occur in most clinical cases, remain largely unknown. Due to inconsistent results brought by the varieties of PUFAs, we crossed *fat-1* mice, which convert n−6 PUFAs to n−3 PUFAs endogenously, with *APC*^Min/+^ mice, generating a novel *fat-1*-*APC*^Min/+^ double transgenic mice model to study the effects of endogenous n−3 PUFAs on colorectal carcinogenesis associated with *APC* mutation. In addition, to simulate tumor development of colorectal cancer associated with *APC* mutation and determine the inhibitory effect of n-3 PUFAs *in vitro*, we constructed a new study model by transfecting the normal colon cell line NCM460 with *APC* siRNA. The combined results from *fat-1*-*APC*^Min/+^ mice, CRC cell lines (SW480 and HCT116), and NCM460 cells with silenced *APC* revealed that elevated n−3/n−6 PUFAs ratio through either intracellular conversion of n−6 to n−3, or through n−3 supplementation, can significantly repress the proliferation and promote apoptosis in colorectal cancer cells, and inhibit the formation of intestinal adenomas, thereby preventing the development of colorectal cancer, as well as the accompanying anemia.

It is remarkable that the mTORC1 activity in the tumors of *fat-1*-*APC*^Min/+^ double-hybrid mice significantly decreased compared with *APC*^Min/+^ mice. Accordingly, DHA or EPA treatment inhibited the mTORC1 activity in the CRC cell lines. Moreover, the ectopic expression of *fat-1* suppressed the mTORC1 activity in NCM460 cells transformed by *APC* depletion. Conversely, treatment of CRC cells with rapamycin, an mTORC1 specific inhibitor, demonstrated growth inhibitory and proapoptotic effects similar to n-3 PUFAs. Taken together, these results suggest that mTORC1 inhibition may be correlated with the effects of increased n-3/n-6 ratio on CRC associated with *APC* mutation. How increased n−3/n−6 ratio suppresses mTORC1 activity needs further investigation, although recent data indicates DHA and EPA increase pTEN expression in breast cancer cells [33], which may in turn suppress pI-3k/Akt and subsequently repress the activation of mTORC1.

Numerous epidemiological studies have suggested that the level of serum lipids is strongly correlated with CRC [34–36]. Consistently, *APC*^Min/+^ mice, a classic and widely recognized mouse model of CRC, also suffer severe hyperlipidemia. However, the role of serum lipids in CRC remains unclear. Interestingly, the *fat-1*-*APC*^Min/+^ double transgenic mice demonstrated significantly decreased serum lipid levels. Because recent evidence suggests that the mTORC1 plays a critical role in PPARγ-mediated adipogenesis [19], fat accretion, and lipemia [20], n−3/n−6 ratio elevated intrinsically in *fat-1*-*APC*^Min/+^ mice might lower serum lipids via suppression of mTORC1. In this context, the fact that enforced expression of *fat-1* in 3T3-L1 preadipocytes restored the mTORC1 activity elevated by *APC* depletion and that DHA treatment inhibited adipogenic differentiation of 3T3-L1 preadipocytes potentiated by *APC* depletion, collectively suggesting a possible role of increasing n-3/n-6 ratio in the suppression of mTORC1 activation induced by *APC* depletion and the consequent adipogenesis and lipemia.

In conclusion, using both transgenic mice and cellular models, we have shown that (1) stable elevation of n−3/n−6 ratio via transgenic expression of *fat-1* prevent colorectal carcinogenesis associated with *APC* mutation and alleviate the accompanying anemia and rectal relapse; (2) the inhibition of mTORC1 by increased n−3/n−6 ratio is associated both with the suppression of CRC development itself and with the rebalance of lipid metabolism associated with CRC. These findings provide novel mechanistic insights into the suppressive effects of n-3 PUFAs on CRC and may shed light on the prevention and non-invasive treatment of CRC as well as its associated complications.

## MATERIALS AND METHODS

### Materials

All cell culture reagents were obtained from Gibco BRL Technology (Gaithersburg, MD, USA). Arachidonic acid and DHA were obtained from Cayman Chemical (Ann Arbor, Michigan, USA). Insulin, antibodies against β-actin, and HRP-conjugated anti-mouse and anti-rabbit IgG antibodies were purchased from Sigma (St Louis, MO, USA). Primary antibodies against phospho-S6 (S235/236) and PARP were obtained from Cell Signaling Technology (Danvers, MA, USA). S6, fat-1, phospho-Akt (S473), and Akt antibodies were purchased from Santa Cruz Biotechnology (Santa Cruz, CA, USA). Recombinant adenovirus with or without the *fat-1* gene was produced by cloning *fat-1* cDNA into the RAPAd CMV using the Adenoviral Expression System (Cell Biolabs) (San Diego, CA, USA).

### Cell culture

Cell lines HCT116, SW480, SW620, NCM460, and 3T3-L1 were obtained from ATCC. Cell lines were frozen in bulk upon receipt and were maintained in RMPI-1640 (NCM460), Leibovitz' L-15 (SW480 and SW620), or Dulbecco's modified Eagle's medium (HCT116 and 3T3-L1) supplemented with 10% fetal bovine serum at 37°C, 5% CO_2_, and 95% humidity.

### Cell proliferation assay

Cells (10^4^/well) were plated in 96-well plates in complete medium. After 24 h, serial concentrations of DHA (10–150 μM) were added to the medium for 72 h, with the replacement of DHA every 24 h. Cell viability was assessed using the Cell Counting Kit-8 (WST-8) (Dojindo Molecular Technologies Inc., Kumamoto, Japan), following the manufacturer's instructions. NCM460 cells were transfected with the pST180-fat-1 vector or with the control vector. After 48 h, cell viability was assessed as described above.

### Assessment of cell death and apoptosis

After 24 h of adhesion, cells were serum-starved and cultured for 8 or 12 h in the presence or absence of serial concentrations of DHA (dissolved in basic medium). Proteins were extracted for the immunoblot analysis of cleaved-PARP. Cells from the 12-h group were trypsinized to make a single cell suspension. A 10-μL volume of 0.4% trypan blue dye was added to a 10-μL volume of cell suspension, and cell death was quantified using a TC10™ Automated Cell Counter (Bio-Rad) according to the manufacturer's instructions. Data from three repeat counts were averaged. Apoptotic cells were also detected with the Annexin V-FITC double-staining kit (BD Pharmingen). For analysis of the apoptotic populations of SW480 cells, both early and late apoptotic cells were scored.

### siRNAs and plasmids transfection

Plated NCM460 and 3T3-L1 cells were transfected with *APC* siRNA or pST180-fat-1 *vector* (see Table for sequences) using Lipofectamine 2000 (Life technologies) according to the manufacturer's instructions. After 48 h, proteins were extracted for western blot analysis.

### Mouse breeding

*APC*^Min/+^ mice (Jax Number: 002020) (C57BL/6 substrain) were bred with *fat-1* transgenic mice (C57BL/6 substrain) to generate *fat-1*- *APC*^Min/+^ double-hybrid mice. Littermates that lacked the *fat-1* transgenic gene were used as controls. DNA extracted from the tails of the offspring were subjected to PCR for genotyping in accordance with the protocol described on the Jackson Laboratory webpage and using primers listed in the Supplementary Method. At 24 weeks of age, the mice were killed by cervical dislocation.

### Tissue harvesting and tumor analysis

Body weight was monitored weekly. After 24 weeks, mice were sacrificed by cervical dislocation under anesthesia with isofluorane (IsoFlo, Burns Vet Supply) and blood samples were collected by intracardiac puncture and immediately stored at −80°C. The entire gastrointestinal tract was immediately removed, washed with phosphate buffered saline (PBS), and divided into four segments [I– III from proximal small intestine (I) to distal small intestine (III), and colon]. The intestines were then spread flat between sheets of filter paper and fixed in buffered 10% formalin for at least 48 h. The number, location, and size of visible tumors were determined at a 10× magnification using an Olympus SZXILLB100 microscope by two independent investigators blinded to the results. Polyps throughout the intestinal tract were classified into four categories (< 1 mm, 1–2 mm, 2–3 mm, and > 3 mm).

### Statistical analysis

Data are presented as mean ± SEM of at least three independent experiments. Differences between groups were analyzed using Student's *t-test* or one-way ANOVA (SPSS version 13.0), and a level of *p* < 0.05 was considered statistically significant. All the experiments including Western Blotting were performed at least three times with similar results.

## SUPPLEMENTARY MATERIALS FIGURES AND TABLES



## Abbreviations

AA: arachidonic acid
APC: adenomatous polyposis coli
CRC: colorectal cancer
DHA: Docosahexaenoic acid
EPA: eicosapentaenoic acid
FAP: familial adenomatous polyposis
mTORC1: mechanistic target of rapamycin complex 1
PUFAs: polyunsaturated fatty acids.
