# Supplementary material for: Elevation of n-3/n-6 PUFAs ratio suppresses mTORC1 and prevents colorectal carcinogenesis associated with APC mutation
Source: Oncotarget. 2016 Oct 19;7(47):76944–54. doi: 10.18632/oncotarget.12759 (PMC5363561; doi:10.18632/oncotarget.12759)
Supplement: Supplementary file 1 [file oncotarget-07-76944-s001.pdf]

Elevation of n-3/n-6 PUFAs ratio suppresses mTORC1 and prevents colorectal carcinogenesis associated with *APC* mutation

Supplementary Materials

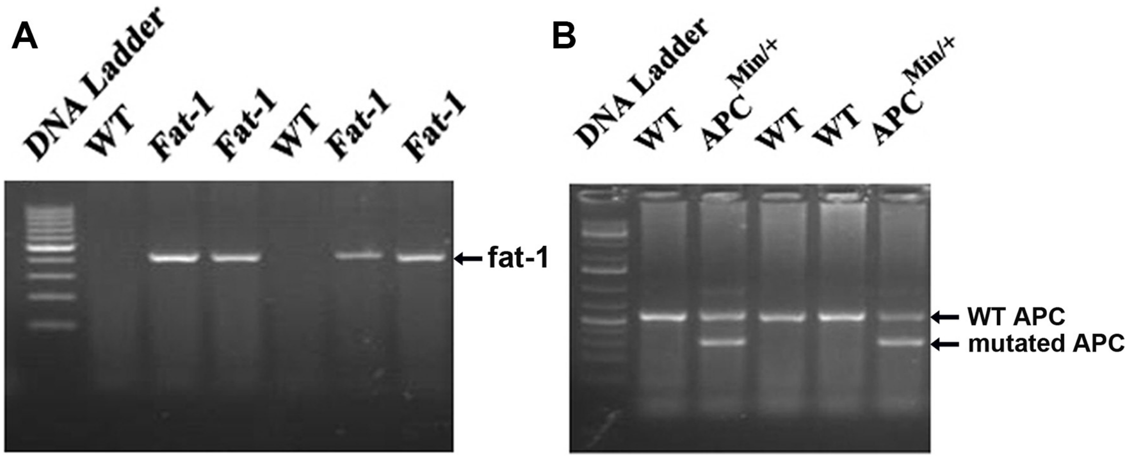

Supplementary Figure S1: Verification of genotype of *fat1-APC*$^{Min+}$ mice. PCR analysis of genomic DNA of *fat1-APC*$^{Min+}$ mice for *fat-1* insertion (left) and APC mutation (right). The PCR product of transgenic *fat-1* is 438 bp, and the product of mutated *APC* is 300 bp, as indicated in the figure.

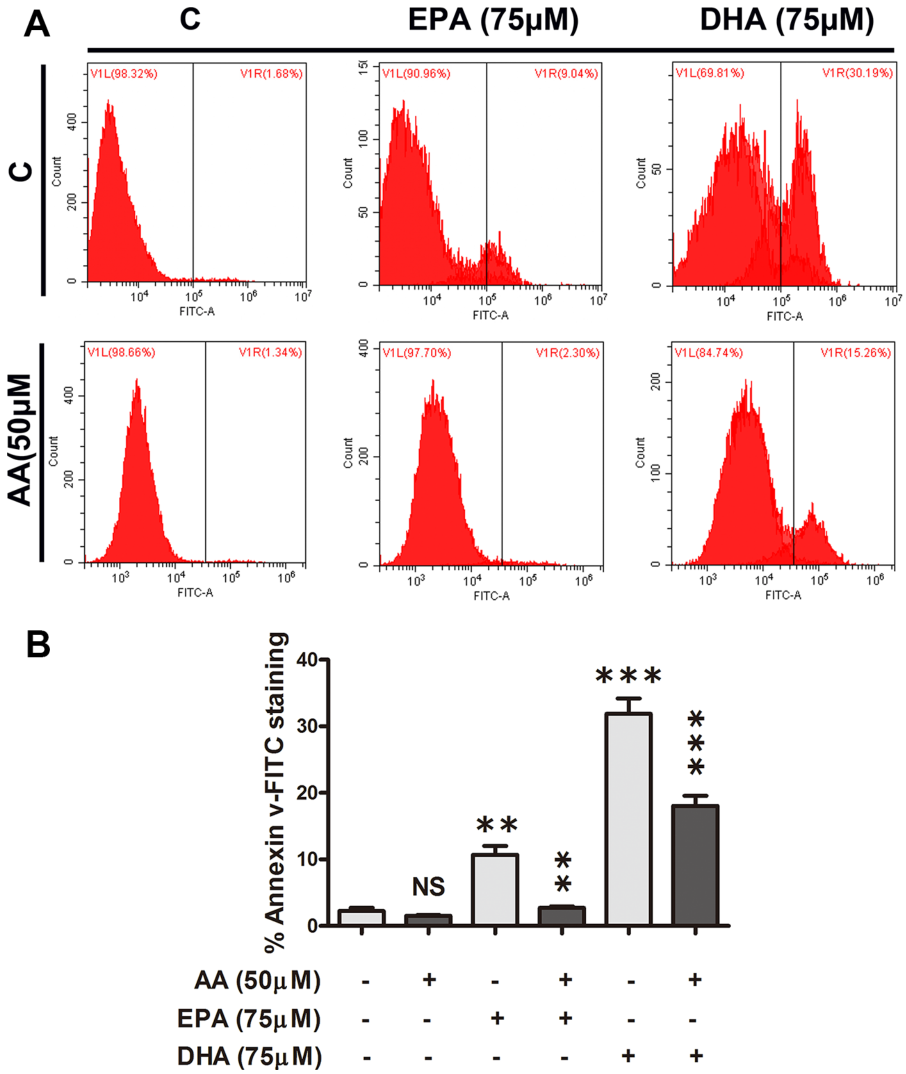

Supplementary Figure S2 (related to Figure 4): DHA or EPA promote apoptosis in SW480 cells. SW480 cells were treated with DHA, EPA, or a combination of AA with DHA (or EPA) for 12 h under serum deprivation, harvested and labelled with Annexin V-FITC and Propidium Iodide (PI). At 10 min post-labelling, apoptotic rate of the cells was determined by flow cytometry. (**A**) Percentages of Annexin V-positive cells (apoptotic cells) are shown from a single representative experiment out of three repeats. (**B**) Statistical data of relative apoptotic rate from three replicates are shown. Horizontal ** or ***, indicates $p < 0.01$ or $p < 0.0001$, respectively VS control (C). Vertical ** or ***, indicates $p < 0.01$ or $p < 0.0001$, respectively VS cells treated the same but without AA.

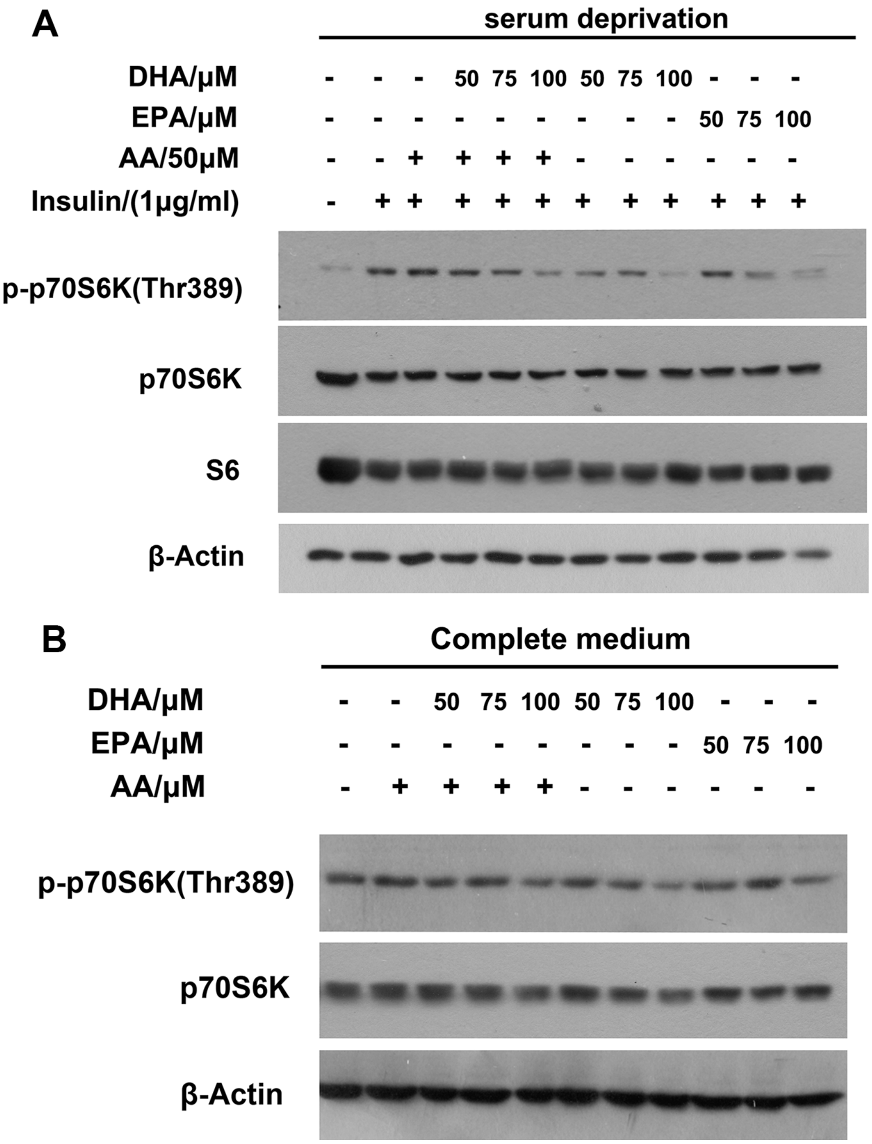

Supplementary Figure S3 (related to Figure 5): DHA or EPA suppresses mTORC1 activity both under serum deprivation and normal growth conditions. (**A**) SW480 cells were treated as indicated under serum deprivation and analyzed for phosphorylation of p70S6K1 (Thr389, one of the best indicators or mTORC1 activity) via Western blotting. Blotting for P70S6K1, S6 and β-Actin were used as loading controls. (**B**) SW480 cells were treated as indicated under normal growth conditions and analyzed as in (A).

Supplementary Table S1: Fatty acids composition in $APC^{Min/+}$ or $APC^{Min/+}$ -fat-1 mouse tails

Type of Fatty Acids (mol % of total fatty acid)	$APC^{Min/+}$	$APC^{Min/+}$ - fat1
C18:3, n−3, α-linoleic acid	0.39 ± 0.05	1.67 ± 0.42
C20:5, n−3, Eicosapentaenoic acid (EPA)	0.08 ± 0.02	0.72 ± 0.13
C22:5, n−3, Docosapentaenoic acid (DPA)	0.31 ± 0.03	0.85 ± 0.26
C22:6, n−3, Docosahexaenoic acid (DHA)	2.00 ± 0.28	2.80 ± 0.39
n − 3, total	2.77 ± 0.31	6.04 ± 0.72
C18:2, n − 6, Linoleic acid	15.48 ± 2.00	10.64 ± 1.42
C20:4, n − 6, Arachidonic acid (AA)	5.43 ± 0.67	0.83 ± 0.32
n − 6, total	20.91 ±2.38	11.47 ± 1.47
n − 3/ n − 6	0.13 ± 0.02	0.53 ± 0.08#

Values of n-3 and n-6 in animal tails (n = 6) were measured by gas chromatography-mass spectroscopy, data indicate mean ± SD. $^{\#}p < 0.01$.

Supplementary Table S2: n−3 and n−6 content in intestinal mucosal scrapings of mice

Type of Fatty Acids	$APC^{Min/+}$	$APC^{Min/+}$ - fat1
C18:3, n −3, α-linoleic acid	0.29 ± 0.03	1.63 ± 0.12
C20:5, n −3, Eicosapentaenoic acid (EPA)	0.09 ± 0.03	0.50 ± 0.19
C22:5, n −3, Docosapentaenoic acid (DPA)	0.32 ± 0.09	0.75 ± 0.19
C22:6, n−3, Docosahexaenoic acid (DHA)	2.18 ± 0.34	3.94 ± 0.51
n − 3, total	2.88 ± 0.39	6.83 ± 0.40
C18:2, n −6, Linoleic acid	16.49 ± 0.75	9.97 ± 1.18
C20:4, n −6, Arachidonic acid (AA)	6.90 ± 0.34	1.24 ± 0.28
n − 6, total	23.40 ± 0.68	11.21 ± 1.19
n −3/ n − 6	0.12 ± 0.01	0.61 ± 0.07#

Values of n−3 and n−6 in intestinal mucosal scrapings of mice (n = 6) were measured by gas chromatography-mass spectroscopy, data indicate mean ± SD. $^{\#}p < 0.01$.

Supplementary Table S3: n−3 and n−6 content in intestinal epithelial cells (NCM460)

Type of Fatty Acids	Vehicle	Fat-1
C18:3, n−3, α-linoleic acid	0.40 ± 0.05	1.49 ± 0.27
C20:5, n−3, Eicosapentaenoic acid (EPA)	0.18 ± 0.06	0.75 ± 0.13
C22:5, n−3, Docosapentaenoic acid (DPA)	0.38 ± 0.05	0.90 ± 0.08
C22:6, n−3, Docosahexaenoic acid (DHA)	2.01 ± 0.60	2.92 ± 0.40
n − 3, total	2.97 ± 0.61	6.07 ± 0.48
C18:2, n−6, Linoleic acid	14.88 ± 0.69	12.96 ± 0.36
C20:4, n−6, Arachidonic acid (AA)	5.95 ± 0.50	1.46 ± 0.21
n − 6, total	20.83 ± 0.96	14.42 ± 0.26
n − 3/ n − 6	0.14 ± 0.03	0.42 ± 0.03[#]

Values of n−3 and n−6 in NCM460 (n = 6) were measured by gas chromatography-masss pectroscopy, data indicate mean ± SD. [#]$p < 0.01$ Vehicle versus Fat-1.
